# Critical Role of Glycogen Synthase Kinase-3β in Regulating the Avian Heterophil Response to *Salmonella enterica* Serovar Enteritidis

**DOI:** 10.3389/fvets.2014.00010

**Published:** 2014-11-24

**Authors:** Michael H. Kogut, Christina L. Swaggerty, Hsin-I Chiang, Kenneth J. Genovese, Haiqi He, Huaijun Zhou, Ryan J. Arsenault

**Affiliations:** ^1^Southern Plains Agricultural Research Center, Agricultural Research Service, United States Department of Agriculture, College Station, TX, USA; ^2^Department of Animal Sciences, National Chung Hsing University, Taichung, Taiwan; ^3^Department of Animal Science, University of California Davis, Davis, CA, USA

**Keywords:** glycogen synthase kinase-3ß, chickens, heterophils, *Salmonella*, innate immunity

## Abstract

A microarray-assisted gene expression screen of chicken heterophils revealed glycogen synthase kinase-3β (GSK-3β), a multifunctional Ser/Thr kinase, to be consistently upregulated 30–180 min following stimulation with *Salmonella enterica* serovar Enteritidis (*S*. Enteritidis). The present study was designed to delineate the role of GSK-3β in regulating the innate function of chicken heterophils in response to *S*. Enteritidis exposure. Using a specific GSK-3β ELISA assay, 30 min after infection with *S*. Enteritidis, heterophils had a significant decrease (*p* ≤ 0.05) in total GSK-3β, but a significant increase (*p* ≤ 0.05) in phosphorylated GSK-3β (Ser9). By 60 min post-infection, there was no difference in the amount of phosphorylated GSK-3β (Ser9) in either the uninfected and infected heterophils. *S*. Enteritidis interaction with heterophils alters GSK-3β activity by stimulating phosphorylation at Ser9 and that peaks by 30 min post-infection. Further, inhibition of GSK3β with lithium chloride resulted in a significant decrease (*p* ≤ 0.05) in NF-κB activation and expression of IL-6, but induces a significant increase (*p* ≤ 0.05) in the expression of the anti-inflammatory cytokine, IL-10. Using a phospho-specific antibody array confirmed the phosphorylation of GSK-3β (Ser9) as well as the phosphorylation of the downstream cytokine-activated intracellular signaling pathway involved in stimulating immune responses, IκB, the IκB subunit IKK-β, and the NF-κB subunits p105, p65, and c-Rel. Our data revealed that the phosphorylation of GSK-3β (Ser9) is responsible for inducing and controlling an innate response to the bacteria. Our findings suggest that the repression of GSK-3 activity is beneficial to the host cell and may act as a target for treatment in controlling intestinal colonization in chickens. Further experiments will define the *in vivo* modulation of GSK-3 as a potential alternative to antibiotics in salmonella and other intestinal bacterial infections.

## Introduction

Polymorphonuclear leukocytes (PMNs) are vital cellular components of innate immunity and function by killing pathogenic microbes following phagocytosis. The primary PMN in poultry is the heterophil, the avian equivalent to the mammalian neutrophil. Like the neutrophil, heterophils provide a rapid deployment of the effector arm of the bird’s innate immune system. Heterophils are rapidly recruited following infection to the site of acute infection. In addition to their well-established role as microbial killers, accumulating evidence shows that heterophils can play an immunoregulatory role (1).

Non-typhoid *Salmonella* infections in poultry induce a rapid acute inflammatory response characterized by an influx of heterophils within hours that, for the most part, restricts infection to the intestine, while activating the innate immune response (2–4). Reducing the number of circulating heterophils significantly increases the susceptibility of young chickens to extra-intestinal infection by *Salmonella enterica* serovar Enteritidis (*S*. Enteritidis) indicating a key effector role for peripheral blood heterophils in controlling acute *S*. Enteritidis infections in poultry (1). Unlike macrophages where *Salmonella* are able to infect and persist, *Salmonella* have not been shown to survive the within heterophils. However, the mechanisms that regulate this antibacterial activity are not understood, although degranulation is considered especially important.

The serine/threonine kinase, glycogen synthase kinase 3β (GSK3β), plays a pivotal role in regulating the inflammatory response of macrophages and neutrophils in mammals (5, 6). GSK3β is unique among kinases in that it is constitutively active in resting cells and its activity can be inhibited by serine phosphorylation by a variety of cellular functions including apoptosis, glycogen metabolism, microtubule function, and cell motility (7, 8). However, it is the enzyme’s ability to regulate elements of both the innate and acquired immune system that has generated the most recent interest (5, 9).

In a recent study involving the whole chicken genome microarray analysis of *S*. Enteritidis-stimulated heterophils, we observed a consistent upregulation of GSK-3β isoform mRNA expression (10, 11). The present study was designed to delineate the role of GSK-3β in regulating the innate function of chicken heterophils in response to *S*. Enteritidis exposure.

## Materials and Methods

### Experimental animals

One-day-old Cobb × Ross straight-run broiler chicks were obtained from a local commercial hatchery and were placed on new pine shavings. Birds were provided water and a balanced, unmedicated ration *ad libitum*. The feed ration contained or exceeded the levels of critical nutrients recommended by the National Research Council (12). All animal experiments were conducted according to the rules and regulations established by the United States Department of Agriculture Animal Care Use Committee and overseen by an attending staff veterinarian.

### Bacteria

A poultry isolate of *Salmonella enterica* serovar Enteritidis (*S*. Enteritidis) (#97-11771) was obtained from the National Veterinary Services Laboratory (Ames, IA, USA). *S*. Enteritidis was cultured in tryptic soy broth (Difco Laboratories, Becton Dickinson Co., Sparks, MD, USA) overnight at 41°C. Stock *S*. Enteritidis [1 × 10^9^ colony forming units (cfu)/ml] was prepared as previously described (13).

### Heterophil isolation

Heterophils were isolated from the peripheral blood of 100 chickens per line 6 days post-hatch. Following blood collection, heterophils were isolated as previously described (14). Briefly, blood from chickens was collected in vacutainer tubes containing disodium ethylenediaminetetraacetic acid (EDTA) (BD vacutainer, Franklin Lakes, NJ, USA) and mixed thoroughly. The blood and EDTA for each line was pooled and diluted 1:1 with RPMI 1640 media containing 1% methylcellulose and centrifuged at 40 *g* for 15 min at 4°C. The supernatant was transferred to a new conical tube and diluted with Ca^2+^- and Mg^2+^-free Hanks balanced salt solution (1:1), layered onto discontinuous Histopaque^®^ gradients (specific gravity 1.077 over 1.119) and centrifuged at 190 *g* for 1 h at 4°C. The Histopaque^®^ layers were collected, washed with RPMI 1640 (1:1), and pelleted at 485 *g* for 15 min at 4°C. The cells were then re-suspended in fresh RPMI 1640, counted on a hemacytometer, and diluted to 1 × 10^7^/ml in RPMI. All tissue culture reagents and chemicals obtained from Sigma Chemical Company, St. Louis, MO, USA, unless noted otherwise.

### Total RNA isolation

Heterophils (1 × 10^7^) were treated with 300 μl RPMI or SE, for 30 and 60 min at 39°C on a rotary shaker at the ratio of multiplicity of infection =20. Treated heterophils were pelleted, washed with RPMI (485 × *g* for 15 min at 4°C), the supernatant discarded, the cells re-suspended in lysis buffer (Qiagen RNeasy mini RNA extraction kit, Qiagen Inc., Valencia, CA, USA), and frozen. The lysed cells were transferred to QIAshredder homogenizer columns and centrifuged for 2 min at ≥8000 × *g*. Total RNA was extracted from the homogenized lysate according to the manufacturer’s instructions, eluted with 50 μl RNase-free water and stored at −80°C. RNA was quantified using a spectrophotometer (NanoDrop Products, Wilmington, DE, USA).

### Microarray experiment design

A dual color, balanced design was used to provide comparisons between uninfected and infected heterophils. Four biological replicates were conducted in each comparison, and the dye balance was used throughout in order to prevent the dye bias during the sample labeling.

#### Labeling and hybridization

The integrity of total RNA samples was confirmed using Agilent Bioanalyzer 2100 Lab-on-chip system (Agilent Technologies, Palo Alto, CA, USA). Five hundred nanograms (ng) of total RNA were reverse-transcribed to cDNA during which a T7 sequence was introduced into cDNA. T7 RNA polymerase-driven RNA synthesis was used for the preparation and labeling of RNA with Cy3 (or Cy5) dye. The fluorescent cRNA probes were purified using Qiagen RNeasy Mini Kit (Qiagen Inc., Valencia, CA, USA), and an equal amount (825 ng) of Cy3 and Cy5 labeled cRNA probes were hybridized on a 44 K chicken Agilent array. The hybridized slides were washed using a commercial kit package (Agilent Technologies, Palo Alto, CA, USA) and then scanned using Genepix 4100A scanner (Molecular Devices Corporation, Sunnyvale, CA, USA) with the tolerance of saturation setting of 0.005%.

#### Microarray data collection and analysis

For each channel, the median of the signal intensity and local background values were used. A locally weighted linear regression (LOWESS) normalization was applied to remove signal intensity-dependent dye bias for each array using R program. The normalized data were analyzed using commercial SAS 9.1.3 program (SAS Institute Inc., Cary, NC, USA) with mixed model analysis. The mixed model used to identify significantly differentially expressed genes was:

$$Y_{\mathrm{ijklm}}\;=\;\mu \;+\;T_{i}\;+\;L_{j}\;+\;D_{k}\;+\;S_{l}\;+\;T\times L_{\mathrm{ij}}\;+\;e_{\mathrm{ijklm}}$$

where *Y*_ijklm_ represents each normalized signal intensity, *μ* is an overall mean value, *T*_i_ is the main effect of treatment (SE infection), *L*_j_ is the main effect of heterophil, *D*_k_ is the main effect of dye, *S*_l_ is the random effect of slide l, *TL*_ij_ is the interaction between treatment and heterophils, and *e*_ijklm_ is a stochastic error (assumed to be normally distributed with mean 0 and variance *σ*^2^). An approximate *F* test on least-square means was used to estimate the significance of difference for each gene in each comparison where *p* < 0.001 was considered to be statistically different. The false discovery rate (*Q* value) was calculated for each *p*-value using R program according to the Storey and Tibshirani method (15).

### GSK-3β ELISA assay

Total and phosphorylated GSK-3β were measured by a solid phase sandwich ELISA kits (Invitrogen, Camarillo, CA, USA). The GSK-3β (total) ELISA kit quantifies GSK-3β independently of phosphorylation status and allows normalization of phosphorylated GSK-3β to total GSK-3β. Preparation of cell extracts was done according to the manufacturer’s instructions. Total amount of GSK-3β (Ser9) was determined using a standard curve.

### Antibody array

The antibody array assay kit was procured from Full Moon BioSystems (Sunnyvale, CA, USA). This technique was used as an alternative to procuring phospho-specific antibodies individually and performing several western blot assays. The protocol was carried out as per manufacturer’s instructions with the following alteration to the homogenization step: instead of using the bead and vortex homogenization indicated in the kit, the hand-held Qiagen TissueRuptor was used.

#### Data analysis for antibody array

Data normalization and PCA analysis was performed for both the peptide and antibody microarrays as per Li et al. (16). This custom analysis method was designed specifically for analysis of phosphorylation microarray data and allowed for a statistically robust analysis of the phosphorylation events being measured. Geneontology (GO) and Kyoto Encyclopedia of Genes and Genomes (KEGG) pathway analysis was performed by uploading the statistically significant peptide lists to the Search Tool for the Retrieval of Interacting Genes (STRING) (17).

### Inhibitor treatments

Heterophils isolated as described above were aliquoted into sterile 2-ml Eppendorf tubes (1 × 10^7^ cells/ml) where they were pre-incubated with the appropriate concentrations of the various inhibitors for 30 min at room temperature. Following these pre-incubations, the heterophils were then stimulated with *S*. Enteritidis (10^9^ cfu/ml) for 1 h at 41°C. The following inhibitors and optimal concentrations were used in these studies: BAY 11-7086 (IκB phosphorylation inhibitor; 50 μM), SN50 (NF-κB inhibitor, 100 μg/ml), and lithium chloride (LiCl, GSK3 inhibitor, 10 mM) as previously determined (18). Based on our previous experiments, the optimal concentrations used in the present experiments had no toxic effects on the avian heterophils.

### Quantitative real-time PCR

Primer and probe sets for the cytokines and 28S rRNA were designed using the Primer Express software program (Applied Biosystems, Foster City, CA, USA). Cytokine and chemokine mRNA expression was quantitated using a well-described method. Primers and probes for cytokines, chemokines, and 28S rRNA-specific amplification have been described (19, 20) and are provided in Table 1. All primers and probes were purchased from Integrated DNA technologies (San Diego, CA, USA). The qRT-PCR was performed using the TaqMan fast universal PCR master mix and one-step RT-PCR master mix reagents [(19, 20); Applied Biosystems]. Amplification and detection of specific products were performed using the Applied Biosystems 7500 Fast real-time PCR system with the following cycle profile: one cycle of 48°C for 30 min and 95°C for 20 s and 40 cycles of 95°C for 3 s and 60°C for 30 s. Quantification was based on the increased fluorescence detected by the 7500 Fast sequence detection system due to hydrolysis of the target-specific probes by the 5 = nuclease activity of the r*Tth* DNA polymerase during PCR amplification. Normalization was carried out against 28S rRNA, which was used as a housekeeping gene. To correct for differences in RNA levels between samples within the experiment, the correction factor for each sample was calculated by dividing the mean threshold cycle (*CT*) value for 28S rRNA-specific product for each sample by the overall mean *CT* value for the 28S rRNA-specific product from all samples. The corrected cytokine mean was calculated as follow: (average of each replicate × cytokine slope)/(28S slope × 28S correction factor). Fold changes in mRNA levels were calculated from mean 40 *CT* values by the formula 2^(40 *CT* infected group − 40 *CT* in non-infected control).^

**Table 1:** **GSK-3β pathway genes from DNA microarray**.

Gene	Description	30′-fold change	*p* value	60′-fold change	*p* value
PI-3K	Phosphatidylinositide 3-kinase	NS	–	NS	–
Akt	Protein kinase B; Serine/threonine protein kinase	NS	–	NS	–
S6 ribosomal protein		NS	–	NS	–
Casein kinase		1.65	0.00012	3.48	1.95 × 10^−10^
Axin-1		3.60	3.9 × 10^−6^	7.29	2.74 × 10^−9^
GSK-3β	Glycogen synthase kinase-3β	3.01	1.72 × 10^−8^	2.69	5.87 × 10^−8^
CREB	*cAMP* response element-binding protein	NS	–	NS	–
IκB	Inhibitor of NF-κB	3.79	9.11 × 10^−11^	4.46	2.13 × 10^−11^
NF-κB	Nuclear factor kappa-light-chain-enhancer of activated B cells	3.34	5.8 × 10^−8^	4.59	3.74 × 10^−9^
IL-10	Interleukin-10	NS	–	NS	–
IL-12 p40	Interleukin-12	2.92 × 10	2 × 10^−5^	28.46	7.89 × 10^−11^
IL-Iβ	Interleukin-1β	16.22	2.59 × 10^−12^	21.04	5.62 × 10^−13^
IL-6	Interleukin-6	9.87	2.39 × 10^−7^	22.73	9.84 × 10^−9^
B-catenin		2.77	2.03 × 10^−5^	2.23	2.55 × 10^−5^

*NS, no statistical differences between uninfected and infected heterophils at the time point assayed*.

*Positive values in fold change mean that genes have a higher expression in infected heterophils*.

*Negative values in fold change mean genes have a higher expression in uninfected heterophils*.

### Degranulation assay

Degranulation was detected by quantifying the amount of β-d-glucuronidase activity in the culture medium following stimulation of the heterophils with *S*. Enteritidis. Heterophils (8 × 10^6^) were incubated with either RPMI 1640 medium alone or the GSK inhibitor, LiCl at room temperature for 30 or 60 min. The heterophils were then stimulated with various MOI of *S*. Enteritidis (1, 10, or 100) for 1 h at 41°C. The reaction was stopped by transferring the tubes containing the cells to an ice bath for 5–10 min. The cells were then centrifuged at 250 *g* for 10 min at 4°C. The supernatants were then removed and used for the assay. A 25 μl aliquot of each supernatant was added to quadruplicate wells in a non-treated, black CoStar flat-bottom ELISA plate and incubated with 50 μl of freshly prepared substrate (10 mM 4-methylumbelliferyl-β-d-glucuronidase, 0.1% Triton X-100 in 0.1M sodium acetate buffer) for 4 h at 41°C. The reaction was stopped by adding 200 μl of stop solution (0.05M glycine and 5 mM EDTA; pH 10.4) to each well. Liberated 4-methylumbelliferone was measured fluorimetrically (excitation wavelength of 355 nm and an emission wavelength of 460 nm) with a GENios Plus Fluorescence Microplate Reader (TECAN US Inc., Research Triangle Park, NC, USA). These values were converted to micromoles of 4-methylumbelliferone generated using a standard curve of known concentrations.

### NF-κB analysis

The ELISA-based Trans-Am transcription factor kit (Active Motif, Carlsbad, CA, USA) was used to detect and quantify NF-κB activation. This kit uses a patented technology to attach oligonucleotides containing an NF-κB binding consensus sequence (5′-GGGACTTTCC-3′) to a 96-well plate according to the transcription factors analyzed (18–20). The active forms of the subunits for NF-κB (p65, p52, p50, c-Rel, RelB) in whole cell extracts can be detected using specific antibodies for epitopes that are accessible only when the nuclear factors are activated and bound to their target DNA. Preparation of cell extract was done according to the manufacturer’s instructions. The specificity of the assays was checked by measuring the ability of soluble wild type or mutated NF-κB oligonucleotides to inhibit binding. The results are expressed as specific binding (absorbance measured in the presence of the mutated oligonucleotides minus that measured in the presence of the wild type oligonucleotides) according to the manufacturer’s instructions.

### Statistical analysis

The anti-coagulated blood from 100 chickens was pooled and the peripheral blood heterophils and monocytes were isolated from each treatment group as described above. Each assay was conducted four times over a 2-month period with pooled cells (heterophils pooled from 100 chickens for each preparation, i.e., 400 chickens in total were used as cell donors). At least three replicates were conducted for each assay with the cells from each pool of chickens. The data from these four repeated experiments were pooled for presentation and statistical analysis.

The mean and standard error of the mean were calculated for each of the treatment groups. Differences between the non-infected and *S*. Enteritidis-infected heterophils were determined by analysis of variance. Significant differences were further separated using Duncan’s multiple range test. The data obtained from the *S*. Enteritidis-infected heterophils were compared to non-stimulated control cells (ANOVA). All statistical analysis was conducted with SigmaStat 3.10 software (Systal Software, Point Richmond, CA, USA).

## Results

### DNA microarray

Concentrating on the canonical GSK-3 pathway from the chicken whole genome array, we found a significant upregulation in the expression of Axin-1, GSK-3β, and β-catenin in the heterophils 30–60 min after infection with *S*. Enteritidis (Table 1). Axin-1, GSK-3β, and β-catenin form an intracellular complex that can regulate multiple signaling pathways involving inflammation (21–23). Further, *S*. Enteritidis infection of the heterophils induced a significant upregulation of IκB, NF-κB, and mRNA of the pro-inflammatory cytokines IL-1β, IL-6, and IL-12 p40 (Table 1). We found no effects on the mRNA expression of the upstream regulators of GSK-3, PI-3K and Akt, or in the expression of the anti-inflammatory cytokine, IL-10.

### *S*. enteritidis modification of GSK-3β phosphorylation

**Figure 1:**
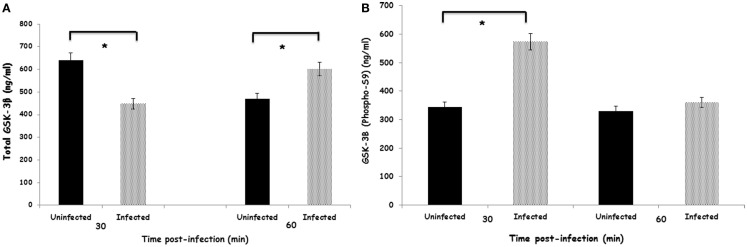
**Effect of *S*. Enteritidis infection on GSK-3β activity in avian heterophils**. **(A)** Total GSK-3β activity in avian heterophils stimulated with *S*. Enteritidis or non-infected for 30 and 60 min. **(B)** Phosphorylated-GSK-3β (Sr9) activity in avian heterophils after 30 and 60 min stimulation with *S*. Enteritidis. Data presented as mean ± SEM from three separate experiments. *p ≤ 0.01.

Within 30 min after infection with *S*. Enteritidis, heterophils had a significant (*p* ≤ 0.05) decrease in total GSK-3β (Figure 1A). By 60 min after infection, the amount of total GSK-3β was reversed where significantly (*p* ≤ 0.05) more total GSK-3β was found in the infected heterophils compared to the uninfected control cells. However, infection of the heterophils with *S*. Enteritidis significantly (*p* ≤ 0.05) increased phosphorylated GSK-3β (Ser9) within 30 min (Figure 1B). By 60 min post-infection, there was no difference in the amount of phosphorylated GSK-3β (Ser9) in either the uninfected and infected heterophils. These data suggest that *S*. Enteritidis interaction with heterophils alters GSK-3β activity by stimulating phosphorylation at Ser9 within 30 min post-infection.

### Role of GSK-3β in cytokine mRNA expression in heterophils infected with *S*. Enteritidis

**Figure 2:**
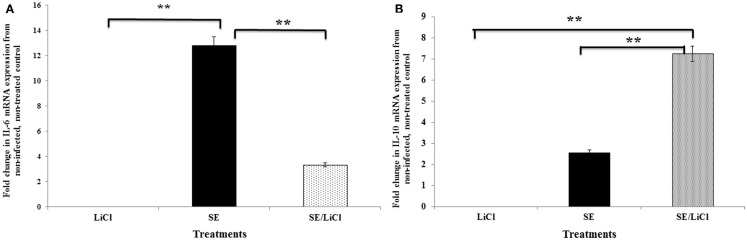
**Inhibition of GSK-3β differentially regulates pro- and anti-inflammatory cytokine mRNA expression by avian heterophils**. Heterophils were pre-incubated with LiCl for 1 h and then stimulated with *S*. Enteritidis (MOI = 100) for 30 and 60 min. The expression of cytokine mRNA was determined by quantitative RT-PCR. Data represent the fold change in mRNA expression in heterophils from infected and/or LiCl-treated groups when compared to the mRNA expression from the non-infected, untreated control heterophils. Data represent the mean ±SEM from three separate experiments. **p ≤ 0.01. **(A)** Expression of IL-6. **(B)** Expression of IL-10.

Stimulation of heterophils with *S*. Enteritidis resulted in increased transcription of the pro-inflammatory cytokine IL-6 (Figure 2A). The expression of IL-6 was significantly (*p* ≤ 0.01) decreased in *S*. Enteritidis-stimulated heterophils pretreated with the specific GSK-3β inhibitor LiCl. Conversely, stimulation of the heterophils with *S*. Enteritidis induced a very limited expression of the anti-inflammatory cytokine, IL-10 (Figure 2B). However, inhibition of GSK-3β by LiCl resulted in a significant (*p* ≤ 0.01) increase in IL-10 mRNA transcription of heterophils stimulated with *S*. Enteritidis. These data point to the regulation of cytokine mRNA expression in heterophils stimulated with *S*. Enteritidis. It should be noted that heterophil viability was not affected by treatment with LiCl (data not shown). Likewise, treatment of the bacteria with LiCl for 1 h had no effect on growth or viability (data not shown).

### Role of GSK-3β in *S*. Enteritidis activation of NF-κB

**Figure 3:**
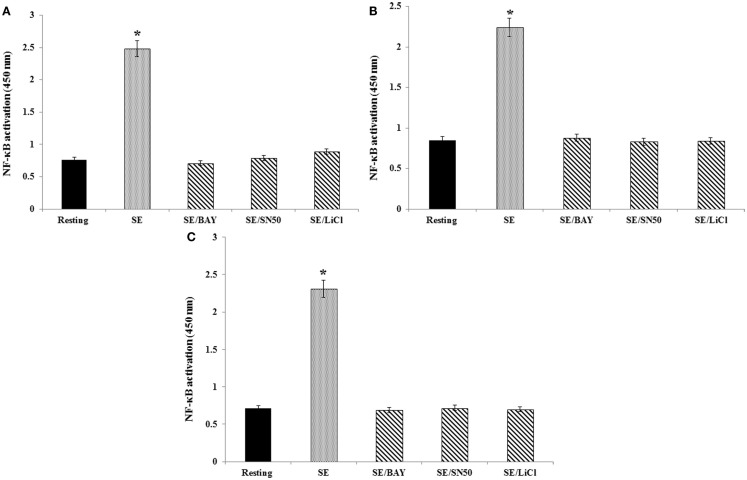
**Effect of NF-κB or GSK-3β inhibitors on binding of either c-Rel, RelB, or p50 subunits to a NF-κB consensus sequence**. Heterophils were aliquoted into sterile 2-ml Eppendorf tubes (1 × 10^7^ cells/ml), where they were pre-incubated with the appropriate concentrations of the various inhibitors for 30 min at room temperature. Following these pre-incubations, the heterophils were then stimulated with *S*. Enteritidis (10^9^ cfu/ml) for 1 h at 41°C. The following inhibitors and optimal concentrations were used in these studies: BAY 11-7086 (IκB phosphorylation inhibitor; 50 μM), SN50 (NF-κB inhibitor, 100 μg/ml), and lithium chloride (LiCl, GSK3 inhibitor, 10 mM). Cell lysates (10 μg/ml) were used for binding of the activated c-Rel **(A)**, RelB **(B)**, and p50 **(C)** subunits to an NF-κB consensus sequence using the Trans-AM NF-κB ELISA kit. The experiment was performed in the presence of soluble wild type or mutated consensus oligonucleotides. The results are expressed as specific binding and are shown as mean ± SE of triplicate experiments. *p ≤ 0.01

To better understand the mechanisms responsible for the effects of GSK-3β on *S*. Enteritidis-induced cytokine mRNA expression, we assessed the activation of NF-κB through the use of the Trans-AM assay of the c-Rel, RelB, and p50 subunits (Figures 3A–C, respectively). We confirmed that stimulation of the avian heterophils with *S*. Enteritidis significantly (*p* ≤ 0.01) activated the nuclear c-Rel, RelB, and p50 subunits of NF-κB that we previously reported (18). To validate the NF-κB subunit activation, we treated the heterophils with either the specific NF-κB inhibitor BAY 11-7086 or the cell-permeable NF-κB inhibitor SN50 and found a total inhibition of activation of each NF-κB subunit (*p* ≤ 0.01; Figures 3A–C).

To assess the functional role of GSK-3β on *S*. Enteritidis-mediated activation of NF-κB, heterophils were treated with the GSK-3β inhibitor LiCl for 30 or 60 min before infection. Inhibition of GSK-3β significantly (*p* ≤ 0.01) decreased activation of each of the NF-κB subunits (Figures 3A–C). These data imply that GSK-3β regulated *S*. Enteritidis-induced cytokine mRNA expression by activating NF-κB.

### Antibody array

Standard methodology for validating the phosphorylation of protein is the use of phospho-specific antibodies. Normally, western blots using antibodies for specific phosphorylation events are performed to confirm the individual phosphorylation events. We chose a variation of this standard validation process by employing an antibody microarray containing both pan-specific and phospho-specific antibodies. Despite the scarcity of chicken-specific antibodies, the key proteins of interest were relatively well conserved between human beings and chickens, giving us confidence that we would observe significant cross-reactivity from the antibodies.

We have previously shown that stimulation of heterophils with *S*. Enteritidis resulted in increased cellular signaling of PI-3K and Akt (24–26). Using a phospho-specific antibody array, we verified a significant phosphorylation of the up-steam regulators of GSK 3β, PI-3K (Tyr607), and Akt (Thr72) (Table 2). Further, the antibody array confirmed the significant phosphorylation of GSK-3β (Ser9) and the phosphorylation of the downstream cytokine-activated intracellular signaling pathway involved in stimulating immune responses, inhibitor of NF-κB, IκB (Ser23), the IκB subunit IKK-β (Tyr188), and the NF-κB subunits p105 (Ser927), p65 (Ser529), and c-Rel (Tyr 1054) (Table 2). Activated IKK-β phosphorylates IκB, which binds NF-κB to inhibit its function. Phosphorylated IκB is degraded via ubiquitination, thus releasing NF-κB, for entry into the nucleus of the cell where it activates various inflammatory and immune response genes (27).

**Table 2:** **Antibody array**.

Protein names	Fold change in antibody array	*p* value
PI-3K p85 (Phospho-Tyr607)	1.19439	0.007701275
Akt1 (Phospho Thr72)	1.257975108	0.023662291
Akt1 (Phospho-Thr450)	1.400143780	0.008226435
Akt1 (Phospho-Thr246)	1.83942	0.01851747
Akt1 (Phospho-Thr 474)	1.069000	0.046647641
p70S6 Ribosomal protein (Phospho-Ser411)	−1.058166076	0.024709023
p70S6 Ribosomal protein (Phospho-Thr 229)	−1.198203352	0.00514916
Casein kinase 1 (Phospho-Thr321)	1.021481145	0.003365457
GSK-3β (Phospho-Ser9)	1.753216	0.014375
CREB (Phospho 133)	−1.06934158	0.015833103
CREB (Phospho-129)	−1.144177379	0.01822392
STAT3 (Phospho-Ser727)	−1.046664685	0.01141102
STAT5A (Phospho-Tyr694)	−1.354049745	0.04702416
STAT6 (Phospho-Tyr641)	−1.378348682	0.024248987
IκκB (Phospho-Tyr188)	1.120064897	0.004702625
IκB (Phospho-Ser23)	1.078084635	0.008557762
NF-κB p105 (Phospho-Ser927)	1.16871	0.000567265
NF-κB p65 (Phosphor-Ser 529)	1.065544284	0.012505685
c-Rel (Phospho-Tyr 1054)	1.250518061	0.029025748
β-catenin (Phospho-Thr41/Ser45)	1.302534522	0.035787183

### Effect of GSK-3β on heterophil degranulation

**Figure 4:**
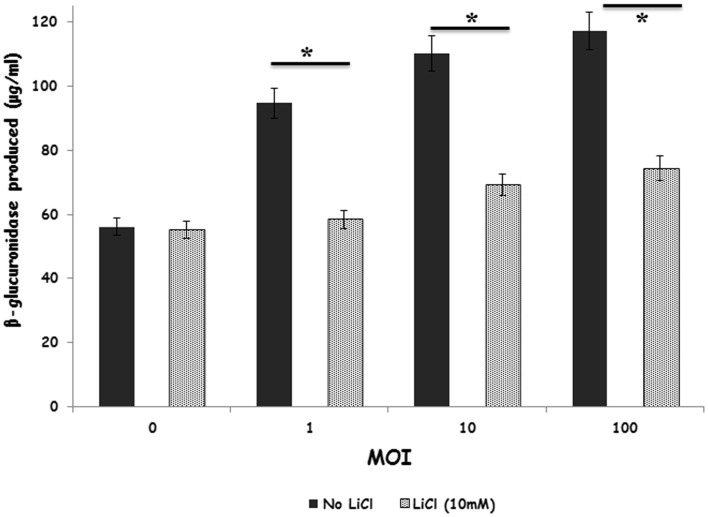
**Inhibition of GSK-3β regulates primary degranulation by avian heterophils stimulated with *S*. Enteritidis**. Heterophils were pre-incubated with LiCl for 1 h and then stimulated with various MOI of *S*. Enteritidis for 1 h at 41°C. Heterophils (8 × 10^6^) were incubated with either RPMI 1640 medium alone or the GSK inhibitor, LiCl at room temperature for 1 h. The heterophils were then stimulated with various MOI of *S*. Enteritidis (1, 10, or 100) for 1 h at 41°C. The reaction was stopped by transferring the tubes containing the cells to an ice bath for 5–10 min. The cells were then centrifuged at 250 *g* for 10 min at 4°C. The supernatants were then removed and used for the assay. A 25 μl aliquot of each supernatant was added to quadruplicate wells in a non-treated, black CoStar flat-bottom ELISA plate and incubated with 50 μl of freshly prepared substrate (10 mM 4-methylumbelliferyl-β-D-glucuronide, 0.1% Triton X-100 in 0.1M sodium acetate buffer) for 4 h at 41°C. The reaction was stopped by adding 200 μl of stop solution (0.05M glycine and 5 mM EDTA; pH 10.4) to each well. Liberated 4-methylumbelliferone was measured fluorimetrically (excitation wavelength of 355 nm and an emission wavelength of 460 nm) with a GENios Plus Fluorescence Microplate Reader (TECAN US Inc., Research Triangle Park, NC, USA). These values were converted to micromoles of 4-methylumbelliferone generated using a standard curve of known concentrations. *p ≤ 0.01.

We verified that stimulation of heterophils with *S*. Enteritidis resulted in increased degranulation in a bacterial concentration-dependent manner (Figure 4). Interestingly, treating the heterophils with the GSK-3β inhibitor, LiCl, significantly (*p* ≤ 0.01) reduced degranulation to baseline levels.

## Discussion

We and others have shown that the heterophil, as the predominant polymorphonuclear cell in poultry, is the primary innate immune effector cell in the initial response to *Salmonella* infection (28–32). Heterophils can be found in the lamina propria of the ceca within hours after *Salmonella* infection (28, 33–36) and are reliant more on degranulation to kill bacteria (37, 38) than an oxidative burst due to the lack of myeloperoxidase (39). Heterophils have been shown to possess all 10 toll-like receptors identified in the chicken, and they can be functionally activated *in vitro* with either TLR agonists or intact bacterial cells (24, 25, 40–44).

We have been investigating the kinase-mediated signaling pathways initiated in heterophils during its interactions with *Salmonella* (24–26, 44), but have not identified the method of regulation of the effector mechanisms within these phagocytic cells. GSK-3 is a multifunctional serine/threonine kinase that has been recently shown to regulate elements of both the innate and acquired immune responses (9, 45). GSK-3 has two major isoforms (α and β) encoded by two separate genes (*gsk3*α and *gsk3*β) (46) that are structurally very similar, but functionally distinct (47, 48). Here, we have characterized the role of GSK-3β in mediating the pro-inflammatory response of heterophils following *S*. Enteritidis infection.

As part of our microarray analysis of *S*. Enteritidis-infected heterophils (10, 11), we observed a significant increase in GSK-3β transcription within 30 min that was maintained at 60 min (Table 1). The differences were also observed at 120 min (3.01-fold change from uninfected controls, *p* < 1.72 × 10^−8^) and 180 min (2.69-fold change from uninfected controls, *p* < 5.37 × 10^−8^). In resting cells, GSK-3 is constitutively active and functions in suppressing multiple cell signaling cascades (49). In the resting cells, GSK-3 forms a complex with the proteins, β-catenin, and adenomatosis polyposis coli (APC) that inhibits the phosphorylation of β-catenin, IκB, and NF-κB and preventing activation of the pro-inflammatory process (8, 45). The increased transcription of GSK-3β in heterophils during their interaction with *S*. Enteritidis implicates its physiological role in the activation of the pro-inflammatory response to infection. Further data found here confirmed this conclusion (increased expression of pro-inflammatory cytokines, decreased expression of anti-inflammatory cytokines, increased heterophils degranulation).

In response to extracellular stimuli, GSK-3 activity can be regulated by phosphorylation (7). GSK-3β can either be activated by phosphorylation of Tyr216 or inactivated by phosphorylation of Ser9. The results found here determined that increased expression of GSK-3β in response to *S*. Enteritidis resulted in an increase in protein level of total GSK-3β (Figure 1A) and that GSK-3β is phosphorylated at Ser9 (Figure 1B). This site-specific phosphorylation of GSK-3β results in the inactivation of its kinase activity (50). Thus, our data imply that heterophil phagocytosis (28) of *Salmonella* specifically alters GSK-3β activity by inducing its phosphorylation.

Transcription factors of the NF-κB family remain in a quiescent state, complexed with inhibitory IκB proteins, in the cytosol of virtually all vertebrate cells. Upon activation, IκB proteins are phosphorylated and released from NF-κB, which then undergoes nuclear translocation and initiates gene transcription (27). NF-κB is composed of homo- and heterodimer complexes made from the five subunits of the NF-κB family (p50, p65, p52, c-Rel, and RelB) (27, 51). The phosphorylation of IκBα following cell activation induces the release of NF-κB dimers, which translocate to the nucleus. In our assays as we described previously (18), we found that *S*. Enteritidis stimulation of heterophils activated NF-κB, composed of the p50, c-Rel, and/or RelB subunits. However, we cannot rule out the activation of either the p65 or p52 subunit during chicken heterophils activation due to unavailability of proper reagents to analyze the role of p65 and p52. Furthermore, treating the heterophils with either the IκB phosphorylation inhibitor, BAY 11-7086, or the cell-permeable, inhibitory peptide of the nuclear translocation of NF-κB, SN50, prevented the activation of NF-κB (Figures 3A–C).

GSK-3β- and NF-κB-mediated signaling pathways are directly linked (52). Inactivation of GSK-3β by phosphorylation at Ser9 induced by bacterial infections results in the degradation of β-catenin, the phosphorylation of IκB by the IKK complex, freeing NF-κB to translocate to the nucleus (53), which, in turn, leads to an increase expression of pro-inflammatory cytokine genes and release of antimicrobial factors. The results of the present experiments confirm that phosphorylation of GSK-3β (Ser9) results in the activation of NF-κB (Figures 3A–C). The role of GSK-3β as a mediator of NF-κB activation was shown by the total inhibition of NF-κB subunit activation by the specific GSK-3β inhibitor, lithium (Figures 3A–C).

As expected and found in previous studies, *S*. Enteritidis infection induced the upregulation of pro-inflammatory cytokines, specifically in these experiments IL-6 (Figure 2A). GSK-3β inactivation using lithium augmented anti-inflammatory cytokine production (IL-10) while concurrently suppressing the production of pro-inflammatory cytokines (Figures 2A,B). Similar results have been described with a number of viral and bacterial infections including Venezuelan Equine encephalitis virus, *Franicisella tularensis*, *Burkholderia psuedomallei*, and *S*. Typhimurium (51, 53–56). It is evident that GSK-3β plays a crucial regulatory role in controlling the quality and extent of the cytokine response to a number of bacterial infections in different hosts. Tay and colleagues (54) have described this as a “distinct survival advantage” for the host during an infection since the event controls the “cytokine storm” that can be initiated and mediated by bacteria during an acute infection as a strategy to favor their own survival (57, 58). Further experiments will be needed to determine whether GSK-3β can be modulated to influence susceptibility to infection in chickens.

By using an antibody microarray containing both pan-specific and phospho-specific antibodies instead of western blots, we were able to quantify the phosphorylation events induced in the GSK-3β signaling cascade (Table 2). A number of interesting responses were found by using the phosphor-specific antibody array. First, we confirmed that infection of *S*. Enteritidis by the heterophils induced the phosphorylation of GSK-3β at Ser9. In addition, we showed the phosphorylation of IκB complex, and phosphorylation of multiple subunits of NF-κB confirming activation demonstrated with the NF-κB activation assays (Figures 3A–C). Second, the antibody array provides data that PI3K/Akt signaling inactivates (phosphorylates) GSK-3β to allow the enhanced NF-κB activity resulting in the increased expression of pro-inflammatory cytokines in heterophils following infection with *S*. Enteritidis. Similar results have been reported in macrophages infected with the gram-negative respiratory bacterium, *Burkholderia cenocepacia* (55). We have previously shown that receptor-mediated phagocytosis of *S*. Enteritidis by heterophils activated PI-3K and Akt (24–26). The antibody array data provide new information on the downstream events by which PI3K/Akt modulates heterophils effector function through inactivation of GSK-3β and enhances NF-κB activity. Third, the inactivation of GSK-3β by phosphorylation at Ser9 positively regulated NF-κB activity, but, based on the antibody array data, negatively regulated the transcription factors, cAMP-response element-binding protein (CREB), and signal-transducer and activator of transcription (STAT3, 5A, and 6). These results imply that the phosphorylation of GSK-3β in heterophils differentially regulate transcription factor activity. This differential regulatory activity on transcription factor activation has previously been reported in human monocytes stimulated with TLR agonists (59). Activation of STAT3 and STAT5 are dependent on GSK-3 activation (60), so inactivation of GSK-3β by phosphorylation inactivates STAT 3 and STAT5 as observed here. Fourth, results from the antibody array showed the dephosphorylation of the target of mTORC1, p70S6 ribosomal protein. These results have been reported in monocytes stimulated with LPS, where mTORC1 regulates GSK-3β activity through the activation of the p70 S6 ribosomal protein (45). Inhibition of S6 ribosomal protein phosphorylation blocks GSK-3β activity resulting in increased pro-inflammatory and decreased anti-inflammatory activities (45). The results from the antibody array are suggestive of a similar mechanism at play in the *S*. Enteritidis-infected heterophils. Finally, we measured an increase in phosphorylation of β-catenin, a transcription factor that regulates cell proliferation and inflammation (53). β-catenin is a negative regulator of inflammation acting in a manner similar to IκB in physically binding to NF-κB, thereby preventing its activation (53, 61, 62). *S*. Typhimurium intestinal infection in a murine model induces the phosphorylation of GSK-3β which, in turn, induces the phosphorylation of β-catenin and results in the translocation of NF-κB to the nuclease (53). Based on the results presented here, we speculate that a similar series of events occurs in the *S*. Enteritidis-infected heterophils resulting in the increased expression of pro-inflammatory cytokines and release of pro-inflammatory effector mediators (degranulation). Although further experiments are required to prove this mechanism of heterophils activation, it is reasonable to believe that based on the results found here that this speculation is accurate.

In summary, we have demonstrated a role for GSK-3β in mediating a pro-inflammatory response in chicken heterophils to infection with *S*. Enteritidis. Our data revealed that the phosphorylation of GSK-3β (Ser9) is responsible for inducing and controlling an innate response to the bacteria. We have identified how GSK-3β regulates the pro-inflammatory reactions of heterophils in response to a *Salmonella* infection and characterized the molecular interactions central to this response. Our findings suggest that the repression of GSK-3 activity is beneficial to the host cell and may act as a target for treatment in controlling intestinal colonization in chickens. Further experiments will define the *in vivo* modulation of GSK-3 as a potential alternative to antibiotics in salmonella and other intestinal bacterial infections.

## Conflict of Interest Statement

The authors declare that the research was conducted in the absence of any commercial or financial relationships that could be construed as a potential conflict of interest. The Review Editor Vivek A. Kuttappan declares that, despite having collaborated with author Michael Kogut in the past 2 years, the review process was handled objectively. The Review Editor Amanda Wolfenden declares that, despite having collaborated with author Michael Kogut, the review process was handled objectively.
